# Vaccine mandates for healthcare workers beyond COVID-19

**DOI:** 10.1136/medethics-2022-108229

**Published:** 2022-05-30

**Authors:** Alberto Giubilini, Julian Savulescu, Jonathan Pugh, Dominic Wilkinson

**Affiliations:** 1 Oxford Uehiro Centre for Practical Ethics, University of Oxford, Oxford, UK; 2 Wellcome Centre for Ethics and Humanities, University of Oxford, Oxford, UK; 3 Murdoch Children's Research Institute, Melbourne, Victoria, Australia; 4 Newborn Care, John Radcliffe Hospital, Oxford, UK

**Keywords:** COVID-19, Coercion, Health Personnel

## Abstract

We provide ethical criteria to establish when vaccine mandates for healthcare workers are ethically justifiable. The relevant criteria are the utility of the vaccine for healthcare workers, the utility for patients (both in terms of prevention of transmission of infection and reduction in staff shortage), and the existence of less restrictive alternatives that can achieve comparable benefits. Healthcare workers have professional obligations to promote the interests of patients that entail exposure to greater risks or infringement of autonomy than ordinary members of the public. Thus, we argue that when vaccine mandates are justified on the basis of these criteria, they are not unfairly discriminatory and the level of coercion they involve is ethically acceptable—and indeed comparable to that already accepted in healthcare employment contracts. Such mandates might be justified even when general population mandates are not. Our conclusion is that, given current evidence, those ethical criteria justify mandates for influenza vaccination, but not COVID-19 vaccination, for healthcare workers. We extend our arguments to other vaccines.

## Introduction

During the COVID-19 pandemic, a number of countries introduced stringent measures designed to address vaccine hesitancy. Some countries implemented vaccine mandates in the general population, for example through some form of vaccine passports (eg, in many European countries) or outright fines for the unvaccinated in certain age groups (eg, in Italy). However, many countries, even those that did not have wider schemes or vaccine passports, made COVID-19 vaccination compulsory for healthcare workers (HCWs).

In England, the government initially planned to make COVID-19 vaccination compulsory for HCWs, starting from April 2022. It also launched a consultation on extending the same approach to influenza vaccination. The Department of Health and Social Care stated:

vaccination reduces the risk of infection, which in turn reduces the risk of transmission. The more staff who are vaccinated against flu and against COVID-19, the more likely it will be that vulnerable people in their care are protected; staff themselves will be protected and their colleagues will also be protected.^1^

However, in January 2022, the UK Government reversed its decision: in the light of the milder Omicron variant (and because of worries about loss of staff), the mandate was no longer considered to be proportionate.

COVID-19 vaccination for HCWs is still mandated in many other countries, including in the European Union and the USA. In the USA, 6 states have mandated COVID-19 vaccination for HCWs as a condition of employment, 15 states have mandated either vaccination or testing, and 3 states have mandated either vaccination or testing and masking, although there are differences as to whether the mandate applies to state employees only and which specific healthcare settings it applies to. However, 13 states have banned vaccine mandates from employers.^1^ The federal COVID-19 vaccine mandate applied to all workplaces was blocked by the Supreme Court, but allowed to go ahead for healthcare facilities receiving federal funding.^2^ At the time of writing, in Australia all states have various forms of vaccine mandates for HCWs, as have all Canadian provinces except for, at the moment, Ontario and Quebec. Some European countries, including France and Italy, have made COVID-19 vaccination mandatory for HCWs.

State vaccine mandates for health professionals are a significant departure from prior policy in the UK and elsewhere. Despite well-documented problems of nosocomial transmission of vaccine-preventable illnesses (eg, influenza),^2 3^ prior to the pandemic most countries did not require HCWs to be vaccinated. In the UK, approximately a quarter of HCWs do not receive the seasonal influenza vaccine.^4^

The aim of this paper is to consider the implications of recent experience, policy and debate for future vaccine mandates. Should HCWs be required to have COVID-19 or annual influenza vaccine as a condition of employment? Should this be extended to include new vaccines against nosocomial infection that are released (eg, future norovirus vaccine^5^), or vaccines against new pandemic threats? We will focus on HCWs, although many of the central issues overlap with questions for residential aged care workers. Recent debate and policies around COVID-19 vaccination for HCWs can be used to further discussion around such questions.

We will argue that, given the current situation in early 2022, COVID-19 vaccination should not be mandated for HCWs but influenza vaccination should. We will outline a framework under which vaccination should be mandatory, on the basis of empirically contingent factors: risks for HCWs, benefits for patients, and the effectiveness of less restrictive options. We will also argue that the level of coercion involved does not in itself make vaccine mandates ethically impermissible, but it must be proportionate to the benefits to patients. Importantly, while the principles we adopt would remain constant, the empirical facts and our level of knowledge may be different in the future, so the very same principles might yield different practical implications in different future scenarios.

## Background on vaccine mandates for health or aged care workers

In healthcare settings, it is well established that vaccines significantly contribute to reducing various kinds of healthcare-associated infections, both viral and bacterial. For the latter, vaccines may have a secondary benefit for the problem of antimicrobial resistance by reducing the need for antibiotics.

The two main benefits of vaccination—illness and contagion prevention—are particularly relevant in the case of HCWs, although they provide different types of justification for vaccination requirements.

Preventing illness is obviously directly beneficial to the HCWs themselves. However, it also benefits the health system and the patients indirectly by reducing the risk of shortage of staff in healthcare settings, an ongoing problem during this pandemic. The probability of developing serious illness depends both on individual risk factors (age, underlying health conditions, obesity, previous infection) and degree of exposure. For instance, in the case of COVID-19, HCWs and other so-called ‘essential workers’ are at higher risk of severe illness than workers considered ‘non-essential’.^6^ The direct benefit of immunity is evident, but the indirect benefit is even more widely enjoyed. Particularly where the requirement to isolate is based on positive tests rather on the presence of severe symptoms, the vaccine may be beneficial in preventing staff shortage even in those at low risk of serious illness.

In January 2022, several hospitals in the UK declared ‘critical incident’ status caused by isolation requirements of staff infected with the Omicron variant. In the week before Christmas 2021, the absence rate of National Health Service (NHS) staff was 8%, compared with a 5% average absence rate in winter months pre-COVID-19.^3^ Influenza typically does not pose this problem to the same degree, since there typically is no routine testing for influenza and therefore no isolation requirement for staff. But in some influenza seasons staff shortages can happen as large numbers of staff fall ill and are unable to work. There is also the opposite problem of ‘presenteeism’, where staff with influenza or influenza-like illness attend work, thus increasing the risk of nosocomial infection.^7^ Both problems could in principle be addressed by reducing the risk of infection in HCWs in the first place. With COVID-19, the widespread use of testing combined with isolation requirements and the presence of a highly transmissible variant like Omicron made the risk of staff shortages higher.

The second benefit of vaccination for HCWs is the potential reduction of serious harm to patients. Public Health England estimates that during the first wave of COVID-19 (beginning February to end of July 2020), 20%–25% of COVID-19 hospitalisations may have been nosocomial.^8^ In Scotland, 30% of ‘COVID-19 deaths’, defined as deaths within 28 days of a positive COVID-19 test in 2020, were considered ‘definitive hospital onset’.^9 10^ The real figures may be higher.

However, while COVID-19 infections have obviously attracted a lot of attention in the past 2 years, COVID-19 is by no means an exception. The lack of routine testing for influenza infection makes it difficult to estimate how many patients catch the influenza in hospitals or care homes every year. The number is not insignificant. For instance, genetic sequencing of influenza samples suggested 15% of patients hospitalised with influenza at London University College hospitals during the 2018–2019 influenza season had a nosocomial infection.^11^

Vaccine mandates are typically controversial as they entail limitations of individual liberties for the sake of the collective good. There is a reasonable disagreement about the extent to which one person should be required to do things to prevent harm to others or even to contribute to collective goods from which they themselves benefit.^12^ Different political and ethical sensitivities would weigh individual rights against the collective good in different ways.

However, when it comes to HCWs, liberty-based counterarguments are more difficult to apply. Quite simply, HCWs have an ethical and professional obligation not to harm patients, or to minimise the risk of harm to patients, which other people do not have. What such obligations include—and whether they include vaccination—depends in part on the standards of negligence, as determined by the majority of professional bodies’ opinions. Normally, they do include vaccination or equivalent safeguards. For instance, the Department of Health in the UK recommends health clearance for tuberculosis, hepatitis B and C, and HIV for new HCWs who will perform exposure-prone procedures. The reason is ‘not to prevent those infected with […] viruses from working in the NHS, but rather to restrict them from working in those clinical areas where their infection may pose a risk to patients in their care’.^13^

This approach is typically reflected in the guidance about vaccination for healthcare staff by medical associations. In the UK, the code of Good Medical Practice states: ‘You should be immunised against common serious communicable diseases (unless otherwise contraindicated)’.^14^ The reason offered is the prevention of passing diseases on to patients. Some Australian states, such as Victoria, also introduced vaccination requirements for healthcare staff before the COVID-19 pandemic. But the need to guarantee adequate healthcare by preventing staff shortage due to illness also features in medical codes. For instance, in September 2021, the American Medical Association called for mandatory COVID-19 vaccination of HCWs as it is ‘needed to sustain the health system into the future as we learn to live with COVID-19’^4^.

Thus, a vaccine mandate for HCWs would be consistent with already existing professional requirements based on preventing harm to patients. However, a legally enforced mandate is different from a professional obligation and it requires additional justification. Not every professional obligation is also a legal requirement. In the next section, we provide three criteria that determine the ethical acceptability of vaccine mandates for HCWs: the criteria pertain to (1) risks of the vaccines, (2) benefits of the vaccines and (3) the availability of less restrictive alternatives to achieve comparable benefits. Criteria 1 and 2 are relevant to determining whether the risks of vaccination are proportionate to its benefits. Criteria 2 and 3 are relevant to determining whether the level of freedom restriction involved in a vaccine mandate is proportionate to its benefits.

## Criteria for the acceptability of vaccine mandates for HCWs

### Risk for HCWs

There is an ethical issue about the nature and magnitude of risks that HCWs should be expected or required to take on as a matter of contractual obligation, for the sake of their patients. It is already commonly accepted that HCWs should take on at least *some* additional health risk for the sake of their patients. This includes, for example, some additional risk of being exposed to infectious disease when they treat infected patients (eg, the risk of needlestick injury^15^). The issue at stake is not if this is justified, but how much extra risk is justifiable by contractual and professional obligations.^16^

If we define as *supererogatory* an action that goes beyond ‘the call of duty’, there is a higher bar for what makes a certain risk supererogatory for HCWs than for the rest of the population. There is a presumption that, barring any significant public health reason, individuals should be free to make their own risk assessment, also based on personal values, about any medical intervention. However, professional obligations more frequently trump individual freedoms.

Whether the risks are proportionate depends, in part, on the benefits we can expect to achieve.^5^ In the case of vaccination, the benefits are measured in terms of the impact of vaccination on patients’ safety and healthcare delivery, which largely depends on the vaccine’s capacity to stop infections but also to prevent HCW staff shortage.

### Benefits

Given the special duties of healthcare professionals to prevent harm to patients, even a small reduction of risk in transmission might ground an ethical and a legal obligation to be vaccinated that does not apply to the general population.

When assessing the benefits of mandatory vaccination, two factors need to be considered: first, the magnitude of the public health threat; and second, whether the mandate can reasonably be expected to increase vaccine uptake in a way that actually translates into significant positive outcomes in terms of patients’ health. This depends in large part on how effective vaccines are at preventing infection and transmission.

### Relative expected utility of a mandate compared with less restrictive options

Whether mandates are ethically justifiable, or even ethically required, depends in part on whether less restrictive options exist that can achieve the same goals.

One proposal is to adopt an intervention ladder on the model of the one the Nuffield Council on Bioethics^17^ proposed for public health interventions more generally. On this proposal, a series of policies, from the least to the most restrictive, should be considered or implemented before the vaccine is made mandatory for HCWs. These are summarised in table 1, which is about COVID-19 vaccines but can be extended to any other vaccination for HCWs and which also includes policies more restrictive than mandates.^18^ Importantly, the table is only meant to present the options available and does not imply that they are all ethically justifiable (in particular, of course, forced vaccination).

**Table 1:** Possible vaccination policies for healthcare workers

Policy	Consequences of vaccine refusal
Forced vaccination	Forcible vaccination; using chemical or physical restraint, if required.
Compulsion/penalties	Fines or imprisonment; termination of employment; cancellation of professional registration.
Professional restrictions/conditions	Employment suspended; enforced leave; loss of salary for days not worked; admitting rights suspended; conditions imposed on professional registration, preventing front-line healthcare work.
Redeployment	Redeployment to non-clinical duties; working from home; restriction on direct clinical work with elderly, vulnerable, immunocompromised patients.
Loss of incentives	No access to employee privileges, such as additional paid leave; no access to restricted areas of the health service, such as tea rooms or health club; no professional registration fee discount.
Nudging/libertarian paternalism	Opt-out policies, such as requiring front-line healthcare workers to sign a declinature statement explaining why they are refusing COVID-19 vaccination; reporting vaccination rates across different teams and highlighting underperforming teams to increase rates, making it harder for underperforming teams to say it is not possible to increase rates.
Persuasion	Education campaigns or professional development activities offered (but not mandated) to persuade FHCWs to reconsider.
No intervention	No intervention if the FHCW refuses or declines vaccination.

However, the difference between less and more restrictive options can be a matter of probability of success. A vaccine mandate might not be strictly necessary, but nevertheless be more likely than a nudging policy or information campaign alone to yield the desired outcomes. In other words, applying a principle of least restrictive alternative often means assuming a risk.

A desire to seek the least restrictive alternative is the basis of the proposal for a ‘conditional mandate’. Such a mandate entails temporary redeployment of the HCW refusing the vaccine to non-clinical roles or, if this is not possible, paid leave, with the contract terminated only at the end of such period of leave if the HCW has not changed their mind and redeployment is still not an option.^18^ This minimises the risk of reducing staff shortage while still achieving the goal of protecting patients.

However, the question is not simply one of using the ‘least restrictive alternative’.^17^ The important question is whether the benefit associated with a more restrictive alternative is sufficiently great to justify the greater restriction.

## COVID-19 vaccine mandates? Probably no

HCWs are at a higher risk of COVID-19 infection and death than the general population and COVID-19 has significant workforce implications, particularly without vaccination.^19^ High infection rates among staff have other undesirable consequences, such as an increased fear in people of contracting COVID-19, which can prevent them from attending healthcare settings and lead to higher morbidity and mortality from other diseases because of late or missed diagnoses.^20^

While these considerations weigh in favour of a COVID-19 vaccine mandate for HCWs, there seem to be, in the present context, stronger reasons against. We will see how COVID-19 vaccine mandates fare with regard to the three criteria identified above.

### Risks for HCWs

COVID-19 vaccines are associated with non-trivial risks, and benefits may only marginally outweigh those risks for many HCWs, especially younger ones, given their relatively low risk of serious illness from COVID-19 and the known and unknown risks of the vaccine. For example, current adenovirus vaccines like the Oxford/AstraZeneca and the Johnson & Johnson vaccines come with a small risk of blood clots, which in 2021 led many countries, including the UK, the USA and Australia, to suspend the use of such vaccines for younger age groups. The Australian Government suspended the use of the AstraZeneca vaccine for people under 60 after having estimated a risk of blood clots of 3.1 cases per 100 000 people under 50 and 2.7 cases per 100 000 people aged 50–59 after one dose of the vaccine.^21^ However, a risk of thrombocytopaenia and thrombosis is also present after contracting COVID-19, with a number of studies indicating that the risk (although still low) is higher after COVID-19 infection than after receiving adenovirus vaccines.^22^

The mRNA vaccines come with risk of myocarditis. One study found that such risk, even if small in both cases, for people under 40 may be higher after the mRNA vaccines than after COVID-19 infection (with 15 excess cases per 1 million after two doses of the Moderna vaccine, lower for other vaccines, compared with 10 excess cases per 1 million after COVID-19 infection).^23^ However, postvaccination myocarditis appears to be mostly mild (treated only with anti-inflammatories),^24^ while COVID-19 (even in younger patients) is associated with increased risks of other severe complications. In the USA, the Centers for Disease Control and Prevention CDC estimate that in men aged 18–24 (the youngest age group likely to be working as HCWs and where the risk/benefit of vaccination is most finely balanced), 1 million mRNA COVID-19 vaccination doses would prevent 936 hospitalisations, 215 intensive care unit (ICU) admissions and 13 deaths, while simultaneously leading to 15–18 vaccine-associated myocarditis cases. (In women aged 18–24, mRNA COVID-19 vaccination would prevent 1127 hospitalisations, 93 ICU admissions and 13 deaths, while causing 4–5 myocarditis cases.^25^

These considerations mean that the marginal benefit of vaccines, especially in young age groups, assuming it is present, is very small. Risks are not insignificant compared with the benefits. This by itself does not make COVID-19 vaccine mandates impermissible. However, it suggests that, at least for certain groups, the argument in favour of a vaccine mandate needs to rely in part on ethical obligations to act in the best interest of the patients or in the interest of the healthcare system, even when this entails some potential additional personal risk. The important question is whether these other benefits are sufficiently great.

### Benefits

As detailed above, COVID-19 has posed a public health threat of a significant magnitude. At earlier stages in the pandemic, the potential benefit of a vaccine mandate in terms of preventing serious illness in vulnerable patients was significant. However, it is less clear that still holds, even if we were to assume that a mandate would be extremely effective in increasing vaccine uptake.

First, current COVID-19 vaccines do not seem very effective at preventing transmission, especially with the highly transmissible Omicron variant, although there is some inconsistency across studies and some are more optimistic than others on the transmission-stopping effectiveness of vaccines.^26^ Even with the less transmissible Delta variant, while vaccination reduced the risk of infection, the viral load (which is linked to risk of transmission) of double-vaccinated people who had breakthrough infection was similar to that of unvaccinated ones, suggesting that ‘both can efficiently transmit infection’.^27^ This proved to be true also for asymptomatic individuals.^28^ At the moment, the only way vaccines could have a significant effect at preventing transmission is by preventing people from getting infected in the first place. Yet vaccines do not appear to be very effective at doing that, although again some studies are slightly more optimistic than others.^26^

As for preventing healthcare staff shortage, the benefit of the vaccine very much depends on the policy in place, particularly with regard to testing requirements. If the policy is that staff testing positive are required to stay home, then the vaccines’ benefits are probably negligible, given vaccines’ low effectiveness at preventing contagion. If there is no testing requirement for asymptomatic staff, then the benefit very much depends on vaccines’ effectiveness at preventing symptoms. A recent study has concluded that two doses of either the Pfizer/BioNTech or the Oxford/AstraZeneca vaccine provide limited protection against symptomatic disease caused by Omicron, and a ‘booster’ dose with an mRNA vaccine would increase protection substantially, but only for a short period of time, falling between 39% and 45% after 2 months.^29^ Unless healthcare staff are required to get vaccinated every 2–3 months (which would compound the risks), it seems the benefit of vaccinating them, both in terms of reducing the risk of infecting patients and reducing staff shortages, would be very limited.

Second, the difference in risk of infection between vaccinated and unvaccinated HCWs has been reduced by significant exposure to the virus over the past 2 years. Prior infection with COVID-19 (sometimes called ‘natural immunity’) is comparable to vaccination in terms of effectiveness at reducing virus transmission of the Delta variant, with a benefit that lasts for at least 13 months.^30^ It is likely that many HCWs have immunity to COVID-19 because of previous infection. Now, prior infection does not offer the same level of protection against Omicron as against Delta. One study estimates a protection through previous infection of 92% against Delta and 56% against Omicron.^31^ A rapid analysis commissioned by the UK Government suggests that the rate of Omicron in those who had two vaccinations was 73.4 infections (per 10 000 person days), compared with 60.9 infections (per 10 000 person days) in those who were unvaccinated but had evidence of prior infection.^32^ It is unclear at the moment for how long any ‘booster dose’ would have an effect on protecting individuals from infection—at the moment we have only observed its effect, which is significant (93%), over the period of a few weeks.^33^ However, data from the Office for National Statistics in the UK indicate that in the first week of January 2022 ‘between 94.1% to 96.3% of the adult population would have tested positive for COVID-19 antibodies at or above a higher antibody threshold needed to provide protection from new COVID-19 infections for those who are vaccinated’. While it is difficult to exactly estimate the actual effect of natural immunity after a mass vaccination roll-out, it is quite telling that the percentage of the adult population who received three doses of the vaccine was estimated to be around only 70% when those data where gathered,^34^ which suggests a significant contribution of natural immunity itself. A large study from Sweden found that it takes 767 people with natural immunity being vaccinated to prevent one reinfection during follow-up, and concluded that ‘[t]he risk of SARS-CoV-2 reinfection and COVID-19 hospitalisation in individuals who have recovered from a previous infection remained low for up to 20 months. Vaccination with an mRNA vaccine seemed to further decrease the risk, although the differences in absolute measures were small’.^35^ If such data are confirmed, the marginal benefit of vaccination in terms of reduction of risk of transmission seems, at the moment, too small to justify a mandate.

Granted, natural immunity in a community might itself become more patchy when the disease is endemic and circulates more slowly. If natural immunity stops offering the level of protection it seems to offer now, the case for introducing vaccination requirements for HCWs in order to protect patients will become stronger.

Third, the severity of illness from COVID-19 has reduced, due to changes in the circulating form of the virus, high vaccine uptake in those at highest risk and increasingly more treatments becoming available.

If vaccines are not very effective at stopping transmission and hence do not prevent serious illness in patients, the infringement on individual freedom, as well as the imposition of vaccine risks on certain groups, might not be justified by the collective benefit. This will be even more likely where there are other, less restrictive ways to achieve a similar result.

### Are there less restrictive policies?

There are other less restrictive ways of minimising the risk of HCWs infecting patients with COVID-19 which are arguably more effective than vaccines. Most obviously, these include regular testing of HCWs and use of personal protective equipment (PPE), in particular adequate masks.^36^ Such alternatives come with their own costs. In particular, PPE can be cumbersome for clinicians to wear and could limit clinician–patient communication, which in some cases could result in worse patient care. That such measures are less restrictive does not mean that they are costless and, once again, the task is that of balancing the costs of different alternatives against each other.

Thus, at the moment the benefits of a mandate do not seem to justify the level of freedom infringement and the costs and risks imposed on the vaccinated. Some have also expressed concerns about the potential impact of a COVID-19 vaccine mandate for black and ethnic minority groups.^37^ Within the NHS, the COVID-19 vaccine uptake in this community is lower than in other groups.^38^ Some take this to mean that ‘they will be disproportionately impacted by the government’s policy’,^37^ although it is worth noting that a vaccination requirement could also have an equalising effect in terms of health outcomes *if* it results in higher vaccine uptake among these communities. This is an issue that would require a separate discussion and we are not going to address in this article.

It is worth emphasising at this stage that we are putting forward claims about the ethical justification of a mandate, not about prudential considerations or ethical considerations for getting the vaccine. There can be strong ethical reasons for being vaccinated despite the small risk (eg, minimising the risk of needing scarce health resources in case one gets ill) and there can be good personal or prudential reasons for being vaccinated (eg, in the case of primary carers who would not be able to care for their dependants if they were severely ill for a prolonged time). Here we are only concerned with ethical and professional requirements as they pertain to HCWs.

## Influenza vaccine mandates? Probably yes

Although the focus of public health policies over the last 2 years has been on COVID-19, influenza represents a significant public health threat. For example, in the 5 years before the COVID-19 pandemic in England, influenza caused on average more than 11 000 annual deaths, with over 22 000 deaths in the 2017 and 2018 season.^1^ Most such deaths occur in people over 65 years, as a weaker immune system means that serious complications of influenza such as pneumonia are more likely and the immune response to the vaccine is poorer.^39^ And of course, the elderly and those with significant comorbidities and/or a weak immune system are over-represented in hospitalised patients.

In the 2020–2021 season, 76.8% of front-line HCWs were vaccinated against influenza in England, with uptake in NHS Trusts ranging from 53.0% to 100%.^4^ In the UK, there is no influenza vaccination requirement for HCWs as a state policy. This is in line with many other countries’ policy. For instance, in the USA no state requires influenza vaccination for HCWs as a condition of employment. Some states require employers to offer vaccination to their healthcare employees and some require them to report their employees’ vaccination status, but employees can be exempted for medical or religious reasons in some of these states and for any reason in the others. However, some individual organisations in the USA do require influenza vaccination as a condition of employment.

### Risk

Influenza vaccines have been used for decades. Changes in the circulating strains causing seasonal influenza mean that protection does not last. The vaccine is modified each year, and both live attenuated and inactivated forms are used. Familiarity with the influenza vaccines means that they have a well-characterised safety profile. Side effects are usually mild and more serious side effects are rare. For example, there is a risk of postvaccine Guillain-Barre syndrome of one per million vaccine doses, although the risk of this is probably higher with influenza itself.^40^

### Benefits

On the one hand, seasonal influenza, even in a bad year, causes considerably less serious illness and death than the COVID-19 pandemic (eg, 30 times fewer deaths based on a recent estimate compared with the 2009 influenza).^41^ On the other hand, as we said above, the mean age at death for influenza is lower than with COVID-19, and the fact that it is endemic means that the total impact remains considerable. (When COVID-19 is endemic and everyone has been exposed, this difference will likely reduce, thanks to higher levels of natural immunity, unless the virus mutates significantly.)

A 2016 systematic review found that mandatory influenza vaccination for HCWs (including termination of employment for those who refuse the vaccine) was ‘by far the most effective single intervention’ at increasing vaccine uptake.^42^ Effectiveness remains very high for cases of ‘soft mandates’. In such cases, declination statements explaining reasons for one’s refusal are sufficient grounds for exemption—a form of conscientious objection to vaccination. This seems confirmed by more recent evidence. In a 2020 study, monitoring the long-term effect of a mandate, the increase within a US institution was from 70% premandate to over 98% after the mandate, with the effect lasting over 10 years and a constant decrease over time in requests for exemptions.^43^

Now, the effect of influenza vaccination in preventing symptomatic infection specifically in healthy adults might be very small. A systematic review suggested ‘healthy adults who receive inactivated parenteral influenza vaccine rather than no vaccine probably experience less influenza, from just over 2% to just under 1% (moderate-certainty evidence)’.^44^ Yet the the Oxford Vaccine Group presents data suggesting that in the five influenza seasons preceding the pandemic, the influenza vaccine prevented 15%–52% of influenza cases across all population groups.^45^

However, one problem is that individuals with asymptomatic influenza can transmit the virus, although the role of asymptomatic cases in virus transmission is uncertain.^46^ A recent study in two South African communities across two influenza seasons has shown that asymptomatic cases of breakthrough infection transmitted infection to 6% of household contacts.^47^ It is estimated that 30%–50% of influenza infections can be asymptomatic,^1 48^ but there are significant challenges to studying influenza vaccine effectiveness against asymptomatic infection, in part because they are not captured by existing surveillance networks. However, to the extent that vaccination reduces the risk of infection, the reason to mandate the vaccine for HCWs in order to prevent harm to patients is strong.

Crucially though, we already have evidence suggesting that increasing vaccine uptake among HCWs translates into reduced risk of harm to patients. A meta-analysis^49^ found a 4.4% reduction in all-cause mortality in hospitals that had increased HCW vaccine uptake by offering influenza vaccination to their staff. Estimates based on modelling indicate that 100% vaccination coverage of HCWs in acute care settings could result in a 43% reduction in the risk of infection in hospital patients and 60% reduction in nursing home patients.^50^ It is worth noting, however, that a systematic review found ‘an absence of high quality evidence that vaccinating HCWs against influenza protects people aged 60 years or older in their care on influenza-specific outcomes’.^51^ While this does not negate the previous claims, it certainly supports the need for high-quality evidence to support any future vaccine mandate.

### Are there less restrictive policies?

Less restrictive measures can be beneficial. For example, simply offering vaccines on-site to HCWs has been shown to increase vaccine uptake.^52^ However, as the evidence summarised above indicates, uptake is significantly higher with a mandate than with other measures. Other types of interventions such as increased awareness, educational initiatives and incentives were found individually to have little effect, although their cumulative effect was comparable to that of ‘soft mandates’.^42^ A more recent meta-analysis further confirmed that interventions including multiple actions are more effective than single actions, finding small to moderate increases in uptake for non-mandatory actions and higher increase when these are accompanied by mandatory requirements (including formal opt-out declarations).^53^

As mentioned above, influenza vaccines are not highly effective at protecting against symptomatic infection. Given this, it has been suggested that influenza vaccine mandates might not be worthwhile if the uptake baseline is already over 70%: it has been estimated that the same results in terms of expected reduced infection following a mandate could be achieved by a 2% reduction in ‘presenteeism’, or working while ill,^54^ which would be a less restrictive measure. However, this line of objection assumes that achieving a 2% reduction in presenteeism could be achieved straightforwardly; yet this is far from obvious. Data suggest that 75% of HCWs who develop an infectious illness continue to work,^55^ so even a 2% reduction in presenteeism represents a significant logistical challenge.^56^ Aiming to reduce presenteeism could be a less restrictive way of potentially achieving a comparable benefit to a vaccine mandate, but it may also be less likely to succeed.

To summarise the argument, HCWs have a professional duty to minimise risk of harm to patients. For what we know at the moment, higher influenza vaccine uptake minimises risk of harm to patients, not just by reducing the risk of infection but also by reducing the risk of staff shortages. Vaccines are effective at reducing risk of infection and illness. Finally, influenza vaccine mandates increase vaccine uptake more than less severe measures (either by themselves or in addition to them).

The above analysis suggests that the relevant ethical criteria would justify mandating the influenza vaccine, but not the COVID-19 vaccine for HCWs at the present time, given the characteristics of current vaccines and the current dominant variant (see table 2).^6^

**Table 2:** Comparison between COVID-19 and flu vaccine

Criteria			
Vaccine	Less restrictive alternatives with comparable benefits?	Benefit (at preventing transmission and staff shortage)	Level of risk
COVID-19	Low	Low	Yes
Influenza	Very low	Moderate	No

If this analysis is correct, the current situation, in which many states mandate the COVID-19 vaccine but do not mandate the influenza vaccine for HCWs, appears to be upside down. If vaccine mandates in HCWs are justified (and we believe that they can be), there is at the moment a stronger ethical case for mandating the influenza vaccine than the COVID-19 vaccine.

It is important to emphasise that we are mostly concerned here with the ethical reasons in support of such mandates. We have based our conclusion on those principles and the evidence currently available. The latter is of course subject to change and, if new evidence emerges that significantly affects the empirical landscape, the same ethical principles could very well support different conclusions.

## Are vaccine mandates discriminatory and coercive?

We have assessed the ethical proportionality of a vaccine mandate—balancing risks, benefits and the restrictiveness. However, some might think that mandates in the form of conditions of employment are unjustifiable in principle, regardless of proportionality. In particular, vaccine mandates might be considered unfairly discriminatory and/or excessively coercive.

We will examine these two considerations separately.

In one sense, discrimination means, quite simply, treating different individuals differently. It is not ethically problematic in itself. In another, normative sense, discrimination is often used to mean ‘unfair discrimination’, that is, when individuals are treated differently on the basis of factors that should not be considered relevant. Thus, in the first, unproblematic sense, all conditions in employment contracts exclude from employment individuals who do not fulfil certain requirements. Vaccination requirements are in a sense not different from requirements to (for example) provide evidence of visa status, copies of qualifications, proof of address and police checks, and so on.

The relevant question is whether it is unfair to require vaccination. This would be the case if vaccination were an ethically irrelevant factor for employment, if vaccination status became a ‘protected characteristic’ that should not create any form of disadvantage in employment decisions (in the way, for example, disability and gender are), if individuals could not access vaccines (thus being excluded from jobs for factors beyond their control), or if the level of coercion involved were excessive.

Vaccination status is an ethically relevant factor when it significantly reduces the risk of infection and contagion. Such reduced risk allows HCWs to fulfil their professional responsibility to minimise the risk of harming patients and to benefit them, as well as healthcare systems’ responsibility to provide adequate healthcare by minimising the risks of shortage of staff.

At the moment, vaccination status is not a protected characteristic, although we do not exclude that it might qualify for this (eg, if vaccination status were taken to express one’s core ethical or religious beliefs). This is a question we are not addressing here. We will simply proceed on the assumption that it is not a protected characteristic, bearing in mind that this might well change.

It would be discriminatory to exclude HCWs if they were unable to access a vaccine (because of cost or availability) or because of a true medical exemption. However, in virtually all settings where a mandate might apply vaccines will be easily available (often at no cost) to HCWs. Those with true medical exemptions should be exempted from vaccination requirements, although in such cases alternative precautions should be adopted (eg, the use of masks and regular testing for asymptomatic infection, if available) if necessary to protect patients.

So the question that remains to be addressed is about the level of coercion involved in vaccine mandates for HCWs: are vaccine mandates coercive and, if so, is the level of coercion ethically acceptable?

All conditions in employment contracts ask future employees to choose between their job and whatever is required. For example, HCWs already agree to health and safety requirements such as washing of hands or wearing gloves and masks when providing (certain forms of) patient care. There is no serious question that an HCW who declined to wash hands (or wear masks/gloves) should not be eligible to provide direct patient care. If this is a form of coercion at all, it is an acceptable one. The problem seems to arise when requirements affect health and lifestyle choices which have broader implications affecting one’s life as a whole. In such cases, arguably the level of coercion is higher. Vaccine mandates fall in this category. However, whether they are excessively coercive depends in part on how one understands coercion.

On so-called non-normative accounts, coercion is what reduces the options available to someone by making them unreasonable, given the magnitude of the penalties for non-compliance or, on some accounts and more controversially, the incentives for compliance.^57^ On such views, vaccine requirements of HCWs are coercive because they reduce the options available to those who refuse the vaccines. The degree of coercion involved will depend in part on the extent of the costs associated with giving up the option of a career as an HCW. However, on normative or ‘moralized accounts’,^58^ coercion only occurs when threats of penalties remove options to which individuals have a right to, or in any case that have some particular moral value. In this sense, to the extent that alternative career paths are available, vaccine requirements for HCWs are not coercive: there is no right to become an HCW and there are plenty of professions (or perhaps specialisations within the healthcare profession) that do not require vaccination as a condition of employment. On this view, a vaccine mandate is no more coercive than any other condition of employment because someone can refuse the vaccine and choose another profession.

However, those who adopt non-normative accounts of coercion can claim that mandates are coercive, and they may note that the costs associated with giving up a career as an HCW are extensive. In particular, alternative career paths are not always easily available, as it presumably is not easy for someone to switch career after having been in the profession for some years. This sounds reasonable: the mandate might not be very coercive for someone who needs to decide whether to become a healthcare professional and knows that that option comes with a vaccination requirement, but it can be very coercive for someone who is already a professional (and entered the profession on the understanding that the vaccine they would rather not take was not mandated). Thus, one way to reduce this coercive pressure and strike a balance between individual freedom and patients’ interests is to make vaccination a condition of entry into the profession rather than mandating those already employed, and adopt a conditional mandate if at all possible for those already in the profession. This is because while someone can relatively easily choose a different career path when young and deciding whether to become a healthcare professional, it is way more difficult, or perhaps an unreasonable option, for someone in their 40s or 50s to switch career because of a new condition of employment that they never agreed to. Whether this level of coercion on someone already in the profession is ethically impermissible depends on whether it is reasonable to expect new health professionals to take future, as yet not developed, vaccines. If that is a reasonable expectation, then again the level of coercion does not seem different from the one posed by any other condition of employment that sets future expectations for employees.

In any case, even if a vaccine mandate places no more coercive pressure on an individual than other conditions of employment, some may regard it as more problematic because of the right it over-rides. Arguably, the right to bodily integrity or bodily autonomy (understood as a right to refuse physical interventions on the body) is a fundamental freedom. We might thus only be willing to accept a lower degree of coercive pressure when this right is at stake, compared with the degree of coercive pressure we accept when other lesser claims are at stake. To illustrate, those who take this view may see a vaccine mandate as problematically coercive where mask/glove/handwashing mandate for HCWs would not in so far as the former infringe upon the right to bodily integrity, but the latter would not. If this is the case, then the permissibility of a mandate will turn on the (1) extent of the coercive pressure that we are willing to accept with respect to interventions that interact with the right to bodily integrity, and (2) the degree of coercive pressure involved in a mandate. Our own view is that if different career choices are easily available (and that is a significant ‘if’), a vaccine mandate need not involve a degree of coercive pressure that would entail an infringement of the right to bodily integrity.

Ultimately, there is an ethical balance to be drawn between protecting patients (including their own right to not acquire serious but preventable nosocomial infections) and coercing some healthcare professionals into having a vaccine that they would prefer not to receive. The harm prevented should be great enough and the risks to professionals small enough to make the coercive pressure justified.

## The analogy with conscientious objection

The balance between patient well-being and professional freedom involved in vaccine mandates for HCWs is similar in important respects to the case of conscientious objection in healthcare. The above suggestion of introducing a requirement as a condition of employment may also apply in this context. According to certain views, freedom of conscience is *permissibly* infringed upon when conscientious objection is ruled out by job contracts, as long as there is a reasonably available option to choose a different profession. This is because the right to freedom of conscience is a qualified one. It is legitimate to make employment subject to eligibility criteria that ensure that professional obligations are discharged. On such views, healthcare professionals do not have a right to conscientious objection where that would violate professional duties, including the duty of beneficence and non-maleficence.^59^

Arguably, such view is questionable and indeed many would not agree. However, even those who think that this line is too strong and are in favour of some form of conscientious objection in healthcare tend to think that conscientious objection should be constrained, for example by requiring a duty of referral and a duty to provide the medical intervention in emergency cases.^60^ This is because patients’ medical interest is taken to normally have priority over personal moral views of healthcare professionals.

Similarly, HCWs might be opposed to vaccines on personal moral or religious grounds. However, putting patients’ interests first means that professional and contractual obligations to avoid causing harm potentially trump those personal beliefs. It is not clear that the exemptions that allow health professionals to conscientiously object as long as a colleague is available to provide the contested service can be extended to non-vaccination for HCWs who have close patient contact. Every interaction means an increased risk of infection to the patient compared with a situation where the HCW is vaccinated.

Thus, a conditional mandate with redeployment to non-patient-facing roles (as per the proposal above) would appear to be the closest equivalent.

## Conclusions: implications for vaccine mandates more generally

Recent debates and policy decisions relating to vaccine mandates for HCWs in the setting of the COVID-19 pandemic might be taken to set a clear precedent for future vaccines. In countries that have adopted such a mandate, the door might now be open for wider application. Given political and apparent ethical support for such mandates, it may be inevitable that countries like Australia, USA, and others will review their approach to influenza vaccination in the coming winters. Alternatively, countries that have taken the opposite approach might be seen to have foreclosed this option. For instance, UK’s former Health Secretary Jeremy Hunt expressed^7^ his view that the scrapping of the COVID-19 vaccine mandate will make it hard to justify other vaccine mandates in the future.

However, in this paper we have suggested that while the ethical principles that support vaccine mandates for HCWs remain constant, circumstances and characteristics of viruses, diseases and vaccine differ. The same principles and criteria can justify mandates for certain vaccines and not for others. Here, we have argued that, given current evidence, they justify influenza vaccine mandates, but not COVID-19 vaccine mandate.

This might sound counterintuitive given that the COVID-19 pandemic has certainly caused substantially more deaths and morbidity than seasonal influenza. Current estimates suggest that it has caused 20 million excess deaths—substantially higher than annual influenza deaths (300 000–600 000).^61^ However, for reasons that we have given above, the balance of risks and benefits suggests that an influenza vaccine mandate, but not a COVID-19 mandate, would currently be ethically proportionate. The same considerations would support mandates for HCWs for other vaccines that offer equivalent prevention of harm and at similar risk to individuals, such as hepatitis B.

Mandates should be introduced on a disease-specific and vaccine-specific basis. The problem must be a significant one, the vaccines must be safe and effective at preventing illness and/or transmission, mandatory measures must be superior to less coercive alternatives, and the costs in loss of liberty and risk to health professionals must be proportionate in professional terms to the benefits to patients. In box 1, we provide a proposal for ethically assessing contractual vaccine mandates in healthcare.

Box 1 **A proposal for contractual vaccine mandates for healthcare workers ie**

- *Employment contracts:* the best way to minimise the degree of coercion while protecting patients and minimising the risk of staff shortage would be to include explicit clauses with vaccination requirements into new employment contracts or potentially at medical/nursing school entry.
- *Conditional mandate:* for staff already employed, it would be preferable to use a conditional mandate where staff is redeployed to non-clinical roles until they are vaccinated, if at all possible, and for as long as it does not become disruptive of healthcare delivery or does not impose unfair burdens on vaccinated colleagues.
- *Selective mandate:* mandates should be tailored to the specific risks that specific diseases pose. For example, hepatitis B vaccine mandates could be applied only to those involved in ‘exposure prone procedures’. Other mandates might apply only in settings where health professionals are in contact with patients at higher risk of serious illness.
- *Natural immunity exemption:* natural immunity should be taken into account. Where it can reasonably be estimated to provide immunity comparable to or even superior to that provided by vaccines, vaccination should not be required as a condition of employment. This means that either healthcare workers with natural immunity should be exempted by any mandate that is otherwise ethically justified, or that the mandate should not be in place if natural immunity among healthcare workers is sufficient to protect patients.

In the case of new vaccines, mandates should be avoided at early stages, while risks are unclear, and be replaced by alternatives (such as testing and protecting personal equipment requirements) or other means to encourage uptake, such as some form of incentives (financial or non-financial). In those cases, contractual (conditional) vaccine mandates should only be considered if alternatives do not offer enough protection from nosocomial infection and there is sufficient evidence around their relative safety, bearing in mind that it is reasonable to expect HCWs to take on themselves some higher degree of risk for the sake of protecting patients than the general population.

## Data Availability

No data are available.

## Footnotes

LeadingAge, 22 December 2021, Vaccine mandates by state: who is, who isn’t, and how?, at https://leadingage.org/workforce/vaccine-mandates-state-who-who-isnt-and-how.

CNBC, 13 January 2022, Supreme Court blocks Biden Covid vaccine mandate for businesses, allows health care worker rule, at https://www.cnbc.com/2022/01/13/supreme-court-ruling-biden-covid-vaccine-mandates.html, accessed 3 February 2022.

BBC, 4 January 2022, Covid: Hospital trusts declare critical incidents over staff shortages, at https://www.bbc.co.uk/news/uk-england-59866650.

AMA, AMA calls for mandatory vaccination for health care staff, *GPNN* 2, September 2021, at https://www.ama.com.au/gpnn/issue-21-number-34/articles/ama-calls-mandatory-vaccination-health-care-staff.

We say ‘in part’ because there may be an upper limit on the level of risk that someone can be professionally or legally required to take, no matter how great the benefit. Above the limit, any additional risk of harm can no longer be ethically justified. The upper limit might be difficult to pin down in some non-arbitrary way. However, even if we have an idea of the threshold of justifiable risk HCWs can be exposed to for the sake of the patients, it remains true that whether the risk of harm is proportionate depends on the benefit that we can reasonably expect to result from it.

Our analysis relates to current knowledge, vaccines and variants for COVID-19. Should a more virulent variant appear and more effective vaccines become available, those criteria might very well justify COVID-19 vaccine mandates in the future.

See his point made in a Twitter thread at https://twitter.com/Jeremy_Hunt/status/1488220491374632963?ref_src=twsrc%5Etfw%7Ctwcamp%5Etweetembed%7Ctwterm%5E1488220491374632963%7Ctwgr%5E%7Ctwcon%5Es1_&ref_url=https%3A%2F%2Ftheconversation.com%2Fis-this-the-end-of-the-road-for-vaccine-mandates-in-healthcare-176310.
